# Modeling and Simulation of Crowd Pre-Evacuation Decision-Making in Complex Traffic Environments

**DOI:** 10.3390/ijerph192416664

**Published:** 2022-12-12

**Authors:** Zhihong Li, Shiyao Qiu, Xiaoyu Wang, Li Zhao

**Affiliations:** 1 Department of Transportation, Beijing University of Civil Engineering and Architecture, No. 1 Zhanlanguan Rd., Beijing 100044, China; 2 China Academy of Urban Planning Design, No. 9 Sanlihe Rd., Beijing 100044, China

**Keywords:** pre-evacuation, decision-making, escape time prediction, human heterogeneity, crowd modeling, public safety

## Abstract

Human movements in complex traffic environments have been successfully simulated by various models. It is crucial to improve crowd safety and urban resilience. However, few studies focus on reproducing human behavior and predicting escape reaction time in the initial judgement stage in complex traffic environments. In this paper, a pedestrian pre-evacuation decision-making model considering pedestrian heterogeneity is proposed for complex environments. Firstly, the model takes different obvious factors into account, including cognition, information, experience, habits, stress, and decision-making ability. Then, according to the preference of the escapees, the personnel decision-making in each stage is divided into two types: stay and escape. Finally, multiple influencing factors are selected to construct the regression equation for prediction of the escape opportunity. The results show that: (1) Choices of escape opportunity are divided into several stages, which are affected by the pedestrian individual risk tolerance, risk categories strength, distance from danger, and reaction of the neighborhood crowd. (2) There are many important factors indicating the pedestrian individual risk tolerance, in which Gen, Group, Time and Mode are a positive correlation, while Age and Zone are a negative correlation. (3) The analysis of the natural response rate of different evacuation strategies shows that 19.81% of people evacuate immediately. The research in this paper can better protect public safety and promote the normal activities of the population.

## 1. Introduction

The international economic situation is complex, and the world is facing unprecedented changes in this century. As an international metropolis, Beijing is characterized by a large city scale, diversified population, and many major projects. The sudden public safety accidents and the superposition effect of different disasters in large cities make urban management very complex. Cities show great vulnerability in the face of various uncertain risks. Both the disaster chain [1] and accident chain [2] are extremely complex, and secondary disasters [3] are often induced.

The public safety and resilience improvement of the urban transportation system is an important part of a resilient city. As the travel of urban residents becomes more and more abundant, the safety of densely populated places is widely concerned. Immediate evacuation is usually the safest action that residents can take when encountering an emergency event, such as an earthquake, tsunami, or fire. However, existing studies show that although some residents may choose to evacuate to a safer place immediately, others may have alternative evacuation options. Specific choices include staying in buildings and actively protecting their property, or taking passive refuge in buildings [4]. The choice of escape opportunity mainly depends on the risk intensity of the emergency and the surrounding environment.

In the case of public safety emergencies, improper pre-evacuation decisions may cause escapees a delay in escape opportunity or a wrong choice of escape route [5,6,7], which are among the main factors in building casualties. Building environment and crowd behavior are closely related. Due to the limited space and differences in a facility’s service capacity in crowded public areas, people show diversified emergency responses and escape behaviors [8,9]. The behavior can also make a dense flow of people disordered, or even state mutation, leading to stampede accidents.

Better understanding and predicting of human pre-evacuation behavior are essential to improve building safety and urban resilience. The complexity of pre-evacuation behaviors in a complex traffic environment can be regarded as a manifestation of complexity science. The coupling between groups, the environment, and individuals shows a much higher level, more complex, and more coordinated order as a whole, mixing with the sudden disturbance of limited individuals. The behaviors of evacuation groups play out through local interactions, such as mutual competition and cooperation among members [10]. At the same time, they have the characteristics of emergence, instability, nonlinearity, and uncertainty. On the one hand, people conduct multi-scale scene experiments and reproduce the evacuation process in the laboratory or use a real scene in combination with questionnaires [11,12,13]. On the other hand, by means of traffic simulation modeling, a group of certain individuals can interact and deduce the situation in the virtual environment by simulating the decision-making behavior in the complex system [14,15]. The decision-making behavior rules of people in an emergency are much more complex than ones in experiments or simulations. Nevertheless, when several individuals are combined to form the group, some characteristics of macro group decision-making behavior will appear from the bottom up. These emerging phenomena objectively reflect the real emergency response system in many aspects. People in crowded public places have the characteristics of large group scale, large characteristic differences, complex relationships, and complex and changeable decision-making behavior with a timeline [16].

Predicting escape time is the direct goal of the analysis and application of evacuation judgement. There has been much commercial software to simulate crowd evacuation to determine whether the required safe evacuation time (RSET) is less than the available safe evacuation time (ASET) or not. RSET calculation time is divided into pre-evacuation and escape movement stages. Escape movement time is the time required for personnel to move purposefully from the beginning to a safe place. There have been relatively mature research results and applications. The pre-evacuation time includes cognitive time and reaction time. It specifically refers to the whole process of finding the alarm (or smoke, shouting, etc.), making clue collection decisions, and finally deciding whether to escape, as well as the selection of an initial route of escape. It also includes time for personnel to wander around, spin around, and pick up valuables. Pre-evacuation time plays an important role in various disaster events [17]. For example, in the “9/11” incident, the pre-evacuation time of many people exceeded 17 min, and the pre-evacuation phase time of some evacuees even exceeded 2/3 of the total evacuation time [18,19]. At the same time, similar observations have been made in fire accidents and exercises in Beijing, Shanghai, Hong Kong, Chicago, and other places [20].

Data on pre-evacuation is usually scarce, incomplete, difficult to access, or in a format not supported by the evacuation model. Data collection and expansion are difficult to solve. Gwynne and Boyce first tried to solve the problem of the pre-evacuation data set in the SFPE [21] manual in 2016. They summarized the existing pre-evacuation data into a table with a unified format and collected 76 cases. Cases are divided into nine types according to building types, and some statistical parameters are given with the characteristic description and pre-evacuation data of various cases. Lovreglio R. et al., expanded this set of data in 2018 and collected 2889 data points, including 112 cases [22]. Relying on the database, the pre-evacuation time and frequency distribution can be calculated, which plays a crucial role in the improvement of the evacuation model. However, it is very difficult to collect real data. The scale of the expanded database is still very small, which cannot characterize the evacuation characteristics of different countries and regions. Its classification method also needs to be more scientific and detailed.

In order to solve the problems of small scale and incomplete features of the database, this paper conducts experimental research on the basis of the existing database with the help of the Analog simulation model. Many experiments have been carried out to prove the correctness of the model and ensure that the prediction model can cover more evacuation characteristics. Firstly, the response mechanism of pre-evacuation behavior under the joint action of internal and external factors is systematically analyzed. Then, a prediction model of group decision-making behavior is proposed to estimate the decision-making time and response rate of the crowd before evacuation. Consequently, we provide useful technical support to improve the evacuation efficiency of the crowd and reduce the severity of the emergency.

## 2. Related Works

### 2.1. Pre-Evacuation Time

Pre-evacuation time mainly refers to the time from event occurrence to the beginning of personnel escape, including the individual perception and detection time of danger, as well as the evacuation response decision-making time. In this process, the personnel shall make evacuation decisions based on the danger found, the degree of danger judged, deciding when to escape, and in which direction to escape.

Many studies have shown that pre-evacuation time is an important part of the expected safe escape time (RSET) [23]. Moreover, through the analysis of past emergencies, the pre-evacuation time is directly related to the number of casualties. According to statistics, for different types of buildings, some researchers used full-scale evacuation experiments and real emergency data to quantify the pre-evacuation time [24]. Other researchers have also investigated the external factors (social or physical environmental factors) and internal factors (individual factors of evacuees) affecting the pre-evacuation time [18]. Nevertheless, there is still a lack of quantitative data on the pre-evacuation time [23].

Some scholars have tried different theories and conceptual models to explain the decision-making process and predict the pre-evacuation time [25], and there are primarily three methods to simulate the pre-evacuation time [26]. The first method adopts the determined user allocation method to allocate the pre-evacuation time of individuals or groups in advance, or set it randomly based on statistical analysis [27,28]. In this case, the escapee stays in place in the cognitive response stage; thus, many mature simulation software use this method. The second method relies on the user’s distribution of activity results during the pre-evacuation response. In this case, the escapee continues to carry out the current activity before escape. For each survivor, the duration of each activity is pre-defined. The disadvantage of these two methods is that the behavior of the escapee is only a pre input, not a real prediction by modeling [29].

The third method is based on prediction. The behavior data of this method is practical. In the simulation, the agent performs protective activities consistent with the internal and external influence factors. However, the pedestrian homogeneity assumption of this method does not reflect the uncertainty of behavior, and there is still a certain gap with the actual situation [26,30]. In recent years, several models have been proposed based on predictability [24], but these models are generally based on behavior theory rather than regression of observed data. Therefore, these models lack data support, resulting in very complex model calibration, which is not solved at present.

Among the existing models and traffic simulation tools, the pre-evacuation time is randomly set or fixed value, which cannot precisely predict the pre-evacuation time. Hence, there is a certain blind area in the pre-evacuation link. In view of the important role and complex characteristics of the pre-evacuation stage in the whole evacuation process, it is vital to establish relevant models to effectively predict the pre-evacuation behavior.

### 2.2. Human Behavior in Crowded Places

Scholars have been studying pedestrian simulation in emergencies and have developed various theories of group behavior [7]. During emergency evacuation, pedestrian response is not random movement, but rather a result based on individual social characteristics. The social characteristics include physical, cognitive, and psychological characteristics. These characteristics affect human behavior and action rules under emergency conditions [31,32]. The existing models of human behavior in the evacuation process are mainly divided into three levels: individual, group, and crowd.

Individual behavior research mainly emphasizes the importance of personal knowledge, culture, and experience in predicting human response. In terms of the characteristics of behavior, individual pedestrians usually pursue the maximization of their own interests. Their escape behavior is mainly based on individual goals [33]. Individual response depends on their familiarity with the surrounding environment and their own cognition of the situation’s severity. When pedestrians are in a familiar area, they tend to despise the severity of the state, delay escape, and flee to a familiar destination [34]. The “place script theory” holds that human reactions are determined by their roles and daily habits, forming fixed rules. It judges individual behavior according to human cognition, risk concept, experience, and daily habits. Galea et al., studied individual evacuation behavior under different cultural characteristics; conducted a questionnaire survey on individual pedestrians in England, Turkey, and China; and systematically analyzed the internal relationship between individual cultural characteristics and evacuation response [8].

The research on group behavior mainly focuses on the structure and rules of groups and the impact of groups on evacuation time. Bode et al., believed that the response behavior of the group was different from that of the individual. During the evacuation, pedestrians will look for nearby acquaintances [35], which will lead to the delay of the initial evacuation time and the extension of the group decision-making time. At the same time, if the cohesion of the team is strong, the team members need to reach an agreement on the escape direction during the evacuation process. The discussion process will also be delayed. Aguirre et al., further applied ENT to explain the crowd response in the 1993 World Trade Center explosion, and the results pointed out that social groups and lasting social relations might increase the evacuation time [36]. Johnson and Kuligowski pointed out that the characteristics of group size and internal relationship type would significantly affect population interaction and state judgment [25,37]. According to the empirical study of the Station Nightclub fire, Aguirre et al., found that even in rapidly spreading emergencies, people in danger looked for close companions [36].

The other research on crowd independence behavior mainly focuses on crowd behavior and decision-making. Crowd behavior is a derivative phenomenon. Previous studies on accidents have shown that an increasing sense of crisis may strengthen collective consciousness. The emergence of collective identity will also stimulate people’s social behavior. People’s behaviors influence each other, and strangers help each other [38]. Others analyzed the evolution trend of group behavior and believed that the structural characteristics of groups were the main factors affecting the intensity of behavior. In the aspect of group decision-making, researchers clearly put forward the theoretical framework of seeking information, interpreting the current situation, assessing risk, and formulating strategies in disaster emergencies. Kuligowski et al., studied the crowd behavior in the early stage of the 9/11 World Trade Center event and developed a decision-making behavior model to quantitatively describe the pedestrian evacuation process [26]. Ronchi et al., proposed an evacuation decision-making model, which predicted the crowd state in the early stage of evacuation by simulating the impact effect of common sense, social impact, alarm, and risk perception level [39].

To sum up, current research on the behavior law of individual, group, and group independence has been relatively perfect. However, the research on Group Evacuation decision-making is still in the exploratory stage, especially the decision-making process in the pre-evacuation stage.

### 2.3. Crowd Management and Simulation Tools

The frequent occurrence of high-density pedestrian congestion and stampede accidents worldwide has attracted many scholars to study the movement law and safety management of large-scale pedestrian flow. A. Johansson and D. Helbing analyzed the pilgrimage video of Mecca in Saudi Arabia in 2006 and revealed the occurrence mechanism of the “stop and go” phenomenon and the “turbulence” phenomenon of high-density pedestrians [6]. RIS S.C. Lee and Roger L. Hughes divided crowded stampede accidents into two categories (stampeded death and crowded death), and discussed the mutual extrusion pressure generated by crowded people [40]. In addition, there are high-density population aggregation studies focusing on social management and risk assessment.

There are many commercial pedestrian simulation software and models. According to the description of pedestrian behavior in pedestrian simulation tools and models, they can be divided into the motion model, partial behavior model, and behavior model. Most of them are motion models, which do not consider the impact of the pedestrian’s subjective will on behavior. It only simulates the crowded collision process during movement, mainly including Steps, Pathfinder, EVACNET4, EgressPro, ENTROPY, magnetic model, etc. Because the motion model ignores the characteristics of pedestrian behavior, it cannot reflect normal situations with complex behavior; thus, it is only suitable for simulating simple emergency evacuations.

In some behavior models, the description of pedestrian behavior is introduced by analyzing pedestrian behavior data. They mainly include transcendence, waiting, and response to fire smoke, but the description is simple and does not really predict behavior. These mainly include Simulex, GridFlow, ALLSAFE, PED/PAX, etc. The behavior model defines the behavior characteristics of any simulated pedestrian in detail. The influence of behavior on path selection is added to the model, and the influence of human-human interaction is considered too. However, due to the complexity of the model, only a few pedestrians can be simulated under the same computing power, and the scope of application is limited. In the behavior model, some probability models are used to describe the randomness of pedestrians, such as EvacSim, BGRFAF, E-SCAPE, ASERJ, etc. The other part uses intelligent algorithms to simulate the selection behavior of a variety of people, such as Legion, SimPed, NOMAD, VegAS. However, these models cannot predict the decision-making of personnel in the pre-evacuation stage.

Scholars have made some achievements in crowd organization, management, and simulation. They carried out relevant evaluations based on simulation models and software, and formulated the congestion management system and emergency mechanism. However, the analysis of influencing factors of personnel behavior and the study of the decision-making mechanism in the pre-evacuation stage still cannot meet the needs of safety production and application. The existing simulation tools also need to supplement and improve the prediction reliability.

## 3. Methodology

Pedestrian groups often show differentiated rational behavior in emergencies [13]. Differentiated rationality can be divided into three different states: complete rationality, partial rationality, and irrational behavior. In practical decision-making, differentiated rationality shows that pedestrians have obvious differences in cognition, information, experience, habits, pressure, and decision-making ability [30]. At the same time, it can explain the actual behavior of decision-makers in real life, and meet the subjective and objective constraints and goals pursued by pedestrians with differentiated characteristics [13]. For example, the goal of pedestrians is not clear at the initial stage. In the process of route selection, its change depends on different degrees of rationality, and the decision-making is restricted by many conditions, such as time, space, cost, and so on. Differentiated rational behavior can better show the micro behavior in pedestrian movement [41].

### 3.1. Situation Evolution of Emergencies

In crowded places, pedestrian interactions come into a complex system. In the existing events, pedestrian casualties are mostly from secondary disasters caused by emergencies [32]. The harm of the emergency itself is limited, but the emergency will bring serious harm to the whole system when it spreads rapidly. Only by recognizing the situation evolution mechanism of the pedestrian movement system in crowded places and analyzing and predicting the system can we make scientific and reasonable responses and regulations. The situation evolution of emergencies in crowded public places is a complex dynamic system that is affected by many factors, including the complicated movement behavior of pedestrians [9], the attributes of emergencies [42], the emergency evacuation plan of the management department [43], the complexity of the place environment and the rationality of the facility setting [44], the cognition of escaping pedestrians [45], etc. The situation evolution of an emergency is affected by its own attributes, the emergency rescue effect of government departments, the emergency capacity of the area where it occurs, and the quality of the affected people. It is a comprehensive dynamic system.

The types of public safe emergencies in this paper mainly focus on fire, earthquake, violent terrorist events, and so on. We do not pay more attention to damage from the event, but mainly study the pedestrian escape decision-making behavior under the condition of secondary disasters caused by events. The choice of pedestrian escape time is affected by many factors, but there are four decisive factors. They are personal risk tolerance (PRT), risk categories and strength (RCS), distance from danger (DFD), and reaction of the surrounding crowd (RSC).

### 3.2. Modelling Evacuation Opportunity Decision Process

(1) Maximum likelihood function

The evacuation process is divided into T decision-making periods. It is assumed that the risk utility of the pedestrian subjective judgment in the *t*-th decision-making period is $E_{it}^{sub}$, which is referred to as subjective risk utility for short. The $E_{it}^{obj}$ is the objective risk utility of the events development. The *RE_it_* is the difference between the subjective risk utility and the safety state utility, $E_{it}=E_{it}^{sub}-E_{it}^{obj}$.

In order to describe the choice of pedestrian evacuation decision nodes, it is assumed that the pedestrian’s choice of wait-and-see and immediate evacuation in the *t*-th decision-making period depends on the difference between subjective expected risk and objective risk utility. If the difference is less than the evacuation selection threshold $ECT_{it}$, pedestrians tend to wait and see; otherwise, pedestrians will escape immediately. It is shown in Equation (1).

(1) $$\mu_{it,t\in \left\{1,2,\cdots T\right\}}=\{\begin{array}{c} 0,\;\;\;\;\;\;if\;\;\;\;\;\;RE_{it}<ECT_{it}\\ 1,\;\;\;\;\;\;if\;\;\;\;\;\;RE_{it}\geq ECT_{it} \end{array}$$

where $\mu_{it}$ is the binary response variable of the evacuation decision. When $\mu_{it}=1$, it means that pedestrians feel danger according to their own judgment and immediately escape. When $\mu_{it}=0$, it means that the current danger still does not reach the pedestrian tolerance limit, and the pedestrian is in a wait-and-see state.

The evacuation coefficient $\lambda_{it}$ is defined to represent the ratio of the escape selection threshold to the subjective expected risk utility, λit=ECTitEitsub. Based on the different pedestrian characteristics, the evacuation selection threshold is related to the individual characteristics of pedestrians (see Equation (2)).

(2) $$\lambda_{it}=f_{escape}\left(PRT,a\right)+\varepsilon_{it}\;\;\;\;\;\;\varepsilon_{it}∼MVN\left(0,\sigma^{2}\right)$$

where the first part $f_{escape}\left(\cdot \right)$ is fixed. Vector PRT is the endurance determined by the characteristics of pedestrians, which include: {Age,Gen,Edu,Time,Group,Zone}. The a is the parameter vector to be estimated. The $\varepsilon_{it}$ is the error term and follows the $MVN\left(0,\sigma^{2}\right)$.

According to the observation and analysis, the random term $\lambda_{it}$ meets the conditions of the Probit regression model. The probability of pedestrian i choosing evacuation decision in t-th period is shown in Equation (3).

(3) Pi(t)=P{REit−ECTit≥0}=P{μit(REit−ECTit)≥0}=P{μit(REit−λitEitsub)≥0}=P{μit[REit−(fescape(PRT,a)+εit)Eitsub]≥0}=P{μitε≤μit[REitEitsub−f(PRT,a)]}

Let $R_{it}=RE_{it}-ECT_{it}$, then $R_{it}$ is the utility of the pedestrian evacuation time selection. The evacuation decision probability of pedestrian *i* in the *t*-th decision-making period can be expressed as Equation (4).

(4) $$P_{i}\left(t\right)=\Phi \left(R_{it}\right)=\Phi \left[RE_{it}-ECT_{it}\right]=\Phi \left[\left(E_{it}^{sub}-E_{it}^{obj}\right)-E_{it}^{sub}f\left(PRT,a\right)\right]$$

The decision-making of pedestrians in any period of time is regarded as independent decision-making. The evacuation decision probability of pedestrian *i* in the *t*-th decision-making period can be expressed in Equation (5).

(5) $$P_{i}\left(t\right)=P_{i}\left(\mu_{i1}=0\right)P_{i}\left(\mu_{i2}=0|\left(\mu_{i1}=0\right)\left(\mu_{i2}=0\right)\right)\cdots P_{i}\left(\mu_{it}=0|\left(\mu_{i1}=0\right)\left(\mu_{i2}=0\right)\cdots \left(\mu_{i\left(t-1\right)}=0\right)\right)$$

Then, $P_{i}\left(t\right)$ can be converted as shown in Equations (6) and (7).

(6) $$P_{i}\left(t\right)=P_{i}\left(\mu_{i1}=0\right)\frac{P_{i}\left[\left(\mu_{i2}=0\right)\left(\mu_{i1}=0\right)\right]}{P_{i}\left(\mu_{i1}=0\right)}\frac{P_{i}\left[\left(\mu_{i3}=0\right)\left(\mu_{i2}=0\right)\left(\mu_{i1}=0\right)\right]}{P_{i}\left[\left(\mu_{i1}=0\right)\left(\mu_{i2}=0\right)\right]}\;\cdots \frac{P_{i}\left[\left(\mu_{it}=1\right)\left(\mu_{i\left(t-1\right)}=0\right)\cdots \left(\mu_{i2}=0\right)\left(\mu_{i1}=0\right)\right]}{P_{i}\left[\left(\mu_{i\left(t-1\right)}=0\right)\cdots \left(\mu_{i2}=0\right)\left(\mu_{i1}=0\right)\right]}$$

(7) $$\begin{array}{cc} P_{i}\left(t\right) & =P_{i}\left[\left(\mu_{it}=1\right)\left(\mu_{i\left(t-1\right)}=0\right)\cdots \left(\mu_{i2}=0\right)\left(\mu_{i1}=0\right)\right]\\ & =P_{i}\left(\mu_{it}=1\right)P_{i}\left(\mu_{i\left(t-1\right)}=0\right)\cdots P_{i}\left(\mu_{i2}=0\right)P_{i}\left(\mu_{i1}=0\right)\\ & =P_{i}\left(\mu_{it}=1\right)\overset{t-1}{\underset{n=1}{\prod }}\left[1-P_{i}\left(\mu_{in}=1\right)\right] \end{array}$$

where $P_{i}\left(\mu_{it}=1\right)$ is the probability that pedestrian *i* decides to escape immediately in the *t*-th decision-making period. At this time, the difference between the pedestrian subjective expected risk and the safety state utility has exceeded the evacuation selection threshold, that is $RE_{it}\geq ECT_{it}$. The maximum likelihood estimation is used to estimate the parameters of the model. The likelihood function is shown in Equation (8), where *L* is the total number of people evacuated.

(8) $$L=\overset{I}{\underset{i=1}{\prod }}P_{i}\left(t\right)=\overset{I}{\underset{i=1}{\prod }}\left[P_{i}\left(\mu_{it}=1\right)\overset{t-1}{\underset{n=1}{\prod }}\left[1-P_{i}\left(\mu_{in}=1\right)\right]\right]$$

(2) Utility model for risk and safety state

The subjective expected risk utility estimation model is expressed as Equation (9).

(9) $$E_{it}^{sub}=f^{Sub}=\underset{j}{\sum }a_{it}fac_{itj}^{sub}=Const_{1}+a_{1}PRT_{i,t}+a_{2}RCS_{i,t}+a_{3}DFD_{i,t}+a_{4}RNC_{i,t}$$

(10) $$E_{it}^{obj}=f^{obj}=\underset{j}{\sum }b_{it}fac_{itj}^{obj}=Const_{2}+b_{1}RCS_{i,t}+b_{2}DFD_{i,t}+b_{3}RNC_{i,t}$$

(11) $$\begin{array}{cc} RE_{it} & =E_{it}^{sub}-E_{it}^{obj}\\ & =Const_{3}+a_{1}PRT_{i,t}+\left(a_{2}-b_{1}\right)RCS_{i,t}+\left(a_{3}-b_{2}\right)DFD_{i,t}\\ & +\left(a_{4}-b_{3}\right)RNC_{i,t}\\ & =Const+c_{1}PRT_{i,t}+c_{2}RCS_{i,t}+c_{3}DFD_{i,t}+c_{4}RNC_{i,t}=E_{it}^{sub}\lambda_{it} \end{array}$$

The objective actual risk utility estimation model is expressed as Equations (10) and (11).

The value range of each factor is [0,1]. The stronger the pedestrian’s risk tolerance, the greater the value of $PRT_{i,t}$.The more dangerous the event, the greater the value of $RCS_{i,t}$.The closer to the risk source, the greater the value of $DFD_{i,t}$. The more serious the panic state of the surrounding people, the greater the value of $RNC_{i,t}$.

Where *PRT* factor is the personal attribute of evacuees, which include *Age*, *Gen*, *Edu*, *Time*, *Group*, *Zone*, *Mode*. *Gen* indicates gender, and *Edu* is education. *Time* indicates familiarity with the surrounding environment. *Group* means whether to travel together or not. *Zone* represents the distance from this position. *Mode* indicates the mode of transportation.

The *RCS* factor mainly refers to the intensity of the risk itself on pedestrian decision-making. The *DFD* factor describes the impact of individual distance from risk sources on individual decision makers. The *RNC* factor emphasizes the herd behavior of individual pedestrians. During the development of the situation, individual pedestrians tend to observe the response of the surrounding people, and then judge whether to escape or not. According to the impact of fire and other events, *RCS*, *DFD*, and *RNC* factors are divided into four levels, as shown in Table 1.

According to the principle of acceptable risk difference, take $RE_{it}=ECT_{it}$. By substituting all the above factors into the equation, the expression of the pedestrian escape selection threshold is shown in Equation (12) and Table 2.

(12) $$\begin{array}{cc} ECT_{it}=RE_{it} & =Const+c_{11}Age_{i}+c_{12}Gen_{i}+c_{13}Edu_{i}+c_{14}Time_{i}+c_{15}Group_{i}\\ & +c_{16}Zone_{i}+c_{17}Mode_{i}+c_{2}RCS_{i,t}+c_{3}DFD_{i}+c_{4}RNC_{i,t}+\varepsilon_{it} \end{array}$$

### 3.3. Simulation and Experiment

(1) Basic scene settings

**Figure 1:**
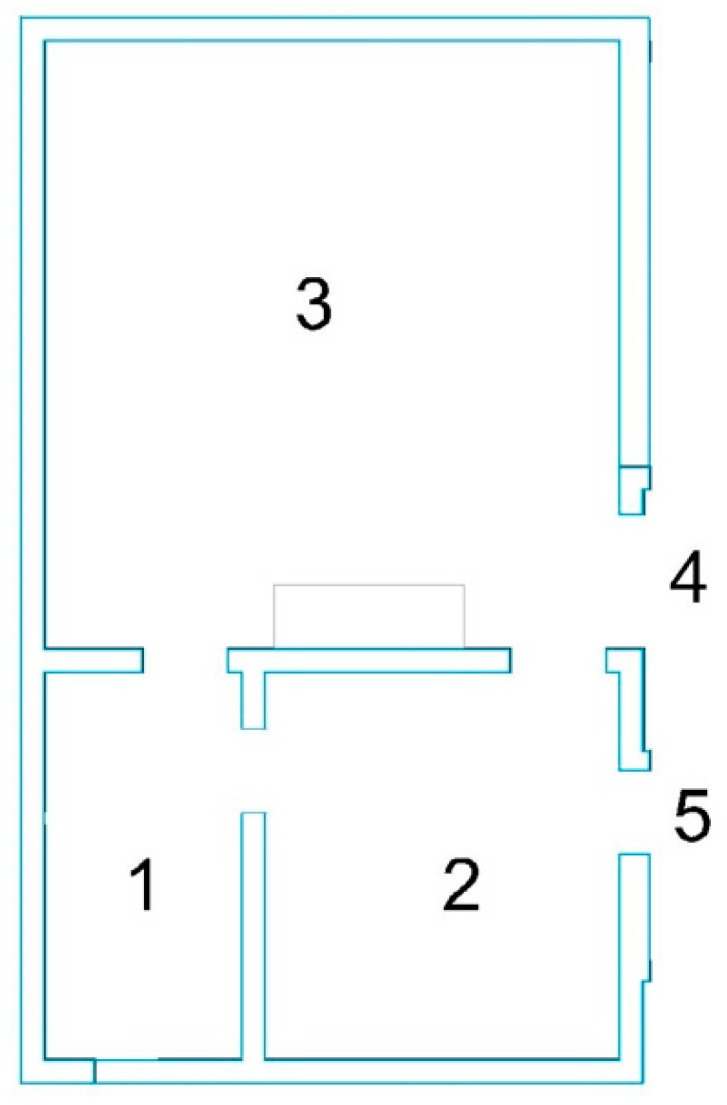
Layout of Architecture. The whole space was divided into three halls, namely 1, 2, and 3. There were entrances and exits between the spaces. The space exits were node 4 and node 5.

In order to verify the effectiveness and calculation process of the personalized exit selection preference model, a simple building layout was selected, as shown in Figure 1. There were 3 evacuation origins (1, 2, and 3, respectively) and 2 evacuation destinations (4 and 5, respectively), as shown in Table 3 and Figure 1.

The timing of the fire was controlled by a fire alarm button inserted in the model. When the fire alarm button was clicked, a fire occurred, and the personnel in the model selected the basic assumptions of the model and the case to evacuate according to the previous evacuation time. The evacuation process is shown in Figure 3. The spread of fire smoke was simulated through the system dynamics model, and the diffusion rate of smoke was set to 0.3 m/s. The yellow area in Figure 4 represents the gradual diffusion of smoke caused by fire.

(2) Individual characteristics and initial layout settings

**Figure 2:**
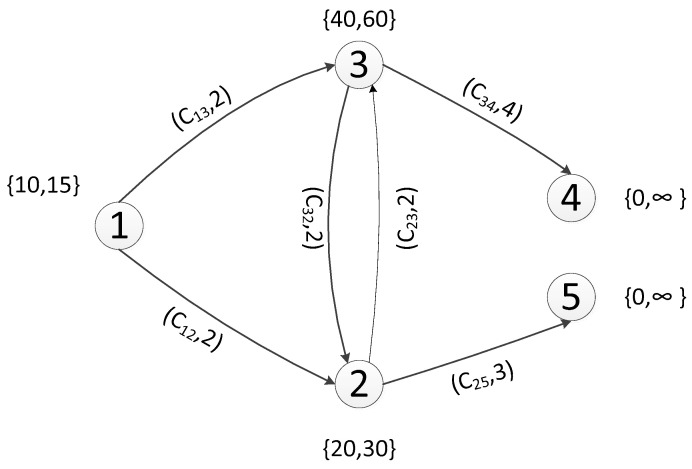
Chart of Net.

**Figure 3:**
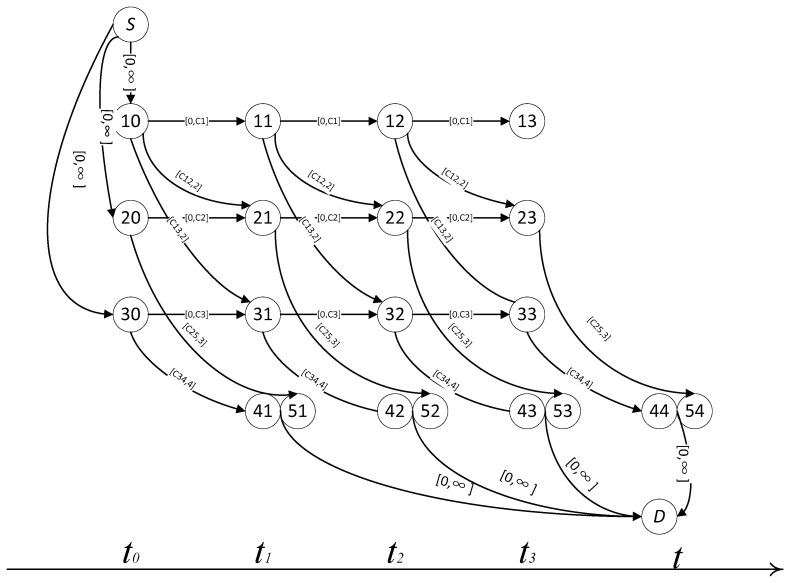
Chart of Multistep Choice in Emergency.

The connection relationship between regions was sampled as shown in Figure 2. Based on the differences of personnel in various areas, different judgments were made on danger and escape time, so different escape behaviors were reflected. The choice of escape time is shown in Figure 3.

**Figure 4:**
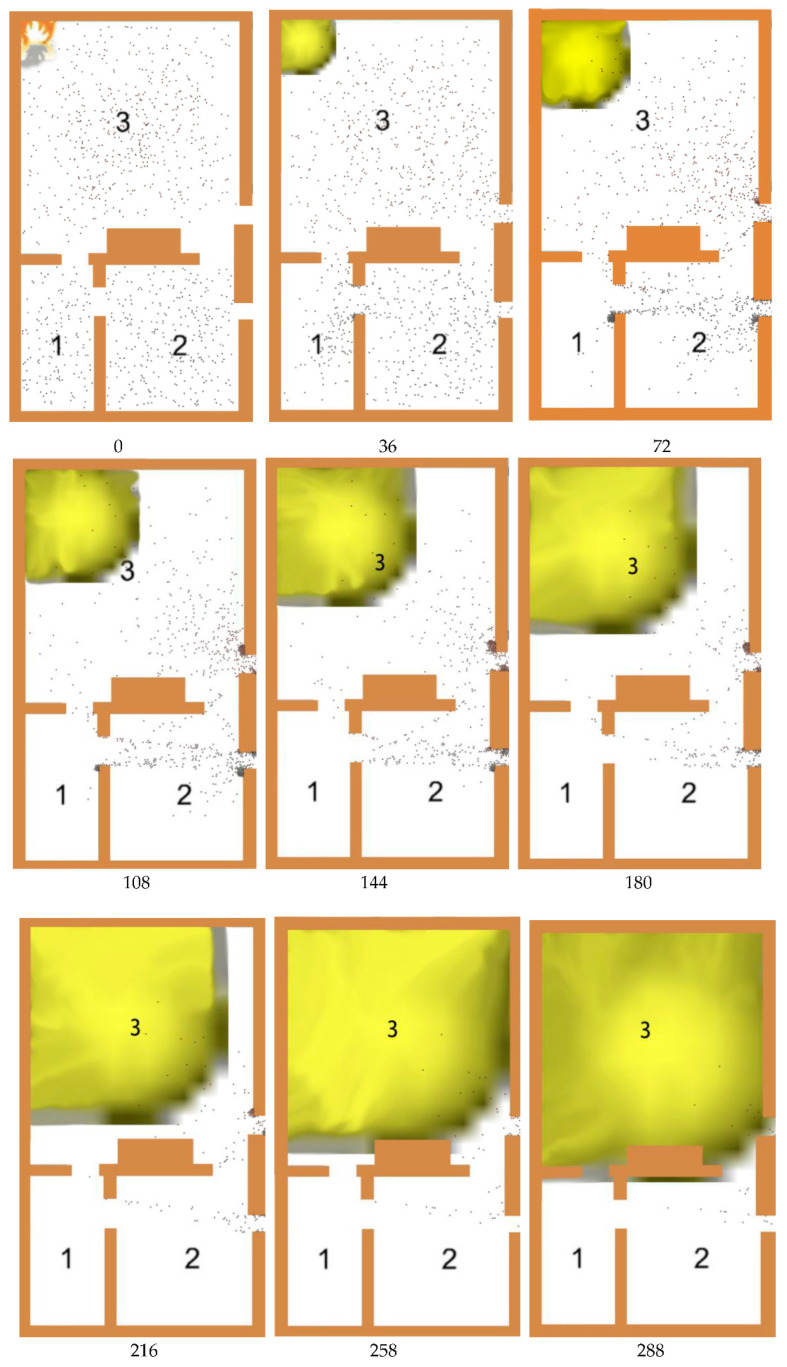
Escape Opportunity Choice in Main Time (Unit: s).

It was assumed that the evacuation demand was distributed in 1, 2, and 3 areas, as shown in Figure 1 and Table 3. The people were randomly distributed in their respective spaces. The individual characteristic attributes were distributed at any time according to the data resources obtained from the questionnaire. Assuming that the fire occurs in Zone 3 and the fire intensity is Level 4, the dynamic evolution process of the fire affected area is shown in Figure 4.

### 3.4. Results and Discussion

According to the previous evacuation time selection model and the basic assumptions of the case, area 3 was a disaster area and personnel received hazard information earlier. Some personnel chose to escape immediately at the initial stage. With the continuation of the event, people in each area began to find the danger and make escape preference decisions.

Figure 4 showed the crowd dynamics at an interval of 36 s in the simulation time period. After the fire, some people in the area close to the fire chose to escape immediately. Most people were still in the wait-and-see state. When a fire occurred, the person closest to the fire would react first. Some people chose to escape quickly, while some people stayed on the sidelines. 

The experiment described the pre-evacuation reaction state caused by fire in the space. When people in different areas found danger, the reaction time was affected by the fire state, the external environment, and personal cognition. When some people around found the danger and did not give warning, others judged the danger according to their own characteristics and decision-making ability. In the face of danger, some people made decisions quickly, while others kept a wait-and-see state. According to the data, the number of people who immediately escaped within 20 s before the evacuation accounted for only 20.1%. The vast majority of people made judgment and analysis, and maintained a wait-and-see state.

In order to analyze the impact of different instructions on evacuation decision-making timing, different types of proposed schemes were used as experimental background factors in the regression process. Regression analysis was performed according to the collected data. Each record represented an observation. The collected data were not the frequency table data combining different value levels of respective variables; the frequency variable count was added to the original data table.

According to the initial construction form of Equation (12), all independent variables were input into covariates. All variables were introduced into the model. If the significance value (Sig.) of individual factors was greater than 0.05, relevant factors would be removed and regression analysis was continued to carry out.

(1) Coefficient calibration

Regression analysis is carried out on the model, and the final parameters in the model are as follows. According to the investigation, the choice of evacuation time is divided into two types (stay or leave) based on the tendency of individual decision-making. According to the response intensity of pedestrians from low to high, the specific variables are expressed as: stay, escape. The two regression models of evacuation timing are shown in Equations (13) and (14), respectively:

(13) $$ECT_{it}^{1}=Const+c_{11}^{1}Age_{i}+c_{12}^{1}Gen_{i}+c_{13}^{1}Edu_{i}+c_{14}^{1}Time_{i}+c_{15}^{1}Group_{i}+c_{16}^{1}Zone_{i}\;+c_{17}^{1}Mode_{i}+c_{2}^{1}RCS_{i,t}+c_{3}^{1}DFD_{i,t}+c_{4}^{1}RNC_{i,t}+\varepsilon_{it}^{1}$$

(14) $$ECT_{it}^{2}=Const+c_{11}^{2}Age_{i}+c_{12}^{2}Gen_{i}+c_{13}^{2}Edu_{i}+c_{14}^{2}Time_{i}+c_{15}^{2}Group_{i}+c_{16}^{2}Zone_{i}\;+c_{17}^{2}Mode_{i}+c_{2}^{2}RCS_{i,t}+c_{3}^{2}DFD_{i,t}+c_{4}^{2}RNC_{i,t}+\varepsilon_{it}^{2}$$

In order to calibrate the relevant parameters, we conducted a questionnaire survey. The questionnaire set up various relevant scene information and personal basic information. A total of 4527 valid questionnaires were collected [46].

Through analysis of the questionnaire data, the parameters calibrated by the pedestrian emergency evacuation decision-making timing model were obtained and shown in Table 4 and Table 5.

(2) Discussion

As listed in Table 4, explanatory variables that had a significant impact on the model include gender (Gen), age (Age), residential area (Zone), number of partners (Group), average arrival times in a month (Time), and mode of transportation (Mode). Meanwhile, educational experience (Edu) was not significant; thus, it was excluded by the model.

The coefficient of Gen was positive, and the escape threshold of men was higher than that of women. It was shown that men are less likely to escape in emergencies. They had a higher probability of watching until the danger reached its threshold point. The coefficient of Age was negative, indicating that the older the age, the lower the escape selection threshold, and the easier it was for pedestrians to escape. The younger the escapers were, the higher the probability of taking a wait-and-see and then escaping. It was related to people’s experience. The older escapers were, the more they tended toward conservative strategies. The coefficient of Group was positive, indicating that the more partners, the higher the escape selection threshold. Pedestrians were less likely to escape early, but had higher expectations of watching. The coefficient of Time was positive, indicating that the more times you appeared, the more conservative you tended to be. Because they were familiar with the surrounding environment, pedestrians who arrived less often chose an earlier escape time. The coefficient of Mode was positive, indicating that the traffic mode was closely related to pedestrian escape.

It was shown that the data fit well with the model, and the significance after the parallel test met the requirements as shown in Table 6 and Table 7. The analysis of the natural response rate of different evacuation strategies showed that under emergencies, 19.81% of the total number of people evacuated spontaneously after abnormal perception, as shown in Table 8.

In the simulation evacuation model, the selection of crowd characteristics and evacuation time was not only an important input parameter, but also a key factor affecting the evacuation simulation results. Due to the particularity of evacuation events, the data resources of evacuation time were very scarce, and the presented data structure was inconsistent with the input results of the evacuation simulation [30]. Although Lovreglio et al. [22] established a database of pre-evacuation times, due to the diversity of the building scenes and evacuation characteristics, there were still many existing scenes to be summarized and supplemented. Based on regression analysis and simulation, the factors affecting evacuation decision-making were selected, among which are age, gender, education level, companionship, and other factors. This was consistent with existing studies [32,33,47]. The response factors of surrounding people are also one of the key factors affecting the timing of escape and evacuation decision-making.

## 4. Conclusions

In this paper, a pedestrian pre-evacuation opportunity decision-making model considering the heterogeneity of evacuees is proposed to make up for the lack of pre-evacuation decision-making environment in public safety evacuation decision-making theory. First, the model considers that there are obvious differences in cognition, information, experience, habits, pressure, and decision-making ability. Then, according to the preferences of the fugitives, the personnel decision-making is divided into two types: stay and escape. Finally, multiple influencing factors are selected to construct a regression equation to predict escape opportunities.

The results show that: (1) Choice of escape opportunity was divided into several periods, which was affected by the pedestrian individual risk tolerance, risk categories and strength, distance from danger, and reaction of the neighborhood crowd. (2) There were many key factors indicating the pedestrian individual risk tolerance, in which Gen, Group, Time, and Mode were positive correlations, while Age and Zone were negative correlations. (3) The analysis of the natural response rate of different evacuation strategies shows that under emergencies, 19.81% of the total number of people evacuate spontaneously and immediately after abnormal perception.

## Figures and Tables

**Table 1:** Standardization of Risk.

	Fire Disaster			Scale
	*RCS*	*DFD*	*RNC*	
L1	Extremely	<20 m	Scream and run	1.00
L2	Great	20–40 m	Organize fire fighting	0.75
L3	Larger	40–60 m	Call the police	0.50
L4	Commonly	>60 m	Wait and see	0.25

**Table 2:** Variable Definition of model.

Variable	Element Definition
$c_{11}\sim c_{16},c_{2}\sim c_{4}$	The parameters to be estimated are obtained according to regression
Const	Constant
$Age_{i}$	Age of pedestrian i. Divided into five grades {<18, 18–30, 31–45, 46–60, >60}, The corresponding values are {1, 2, 3, 4, 5}
$Gen_{i}$	Gender of pedestrian i. {Male, Female}, The corresponding values are {1, 0}
$Edu_{i}$	Education of pedestrian i. {High school and below, College, Graduate and above}, The corresponding values are {1, 2, 3}
$Time_{i}$	Length of time that pedestrians i is familiar with the area. {Less than 2 years, 2–5 years, 5–10 years,10 year above}, The corresponding values are {1, 2, 3, 4}
$Group_{i}$	Pedestrian i companionship, {1 ped, 2 ped, 3 ped, 3 ped and above}, The corresponding values are {1, 2, 3, 4}
$Zone_{i}$	Distance relationship between pedestrian i residence and the area, {<2 km, 2–5 km, 5–10 km, 10–30 km, 30 km above}, The corresponding values are {1, 2, 3, 4}
$Mode_{i}$	Pedestrian i mode of transportation, {Car, Subway, Tricycle, Conventional bus, Bicycle, walk, taxi}, The corresponding values are {1, 2, 3, 4, 5, 6, 7}
$RCS_{i,t}$	It refers to the intensity of risk itself on pedestrian decision-making, which is divided into four levels
$DFD_{i,t}$	Distance between pedestrians and dangerous core in period t. It is divided into four levels
$RNC_{i,t}$	The response of surrounding people in the t period is divided into four levels
$\varepsilon_{it}$	Random term

**Table 3:** Distribution of Escape Demand.

Origins of Evacuation	Evacuation Demand
1	14.3%
2	28.6%
3	57.1%
Total	100%

**Table 4:** Regression of Model Parameters.

	Variable	B	S.E.	Z	Sig.	95% C.I.	
						Lower	Upper
Probit ^a^	Gen	0.011	0.011	0.957	0.034	−0.011	0.032
	Age	−0.015	0.007	−2.156	0.031	−0.028	−0.001
	Edu						
	Zone	−0.013	0.005	−2.446	0.014	−0.024	−0.003
	Group	0.013	0.007	1.728	0.084	−0.002	0.027
	Time	0.004	0.001	3.257	0.001	0.001	0.006
	Mode	0.002	0.003	0.800	0.042	−0.003	0.007
	Interception b						
	1	−3.212	0.034	−93.888	0.000	−3.246	−3.178
	2	−3.185	0.035	−90.681	0.000	−3.220	−3.150

^a^ PROBIT Model: PROBIT (p) = b + BX.

**Table 5:** Covariation and Relationship of Estimated Parameters.

		Gen	Age	Zone	Group	Time	Mode
Probit ^a^	Gen	0.000	−0.001	0.026	0.033	0.003	0.069
	Age	0.000	0.000	−0.046	0.086	−0.288	0.025
	Zone	0.000	0.000	0.000	0.015	0.015	0.235
	Group	0.000	0.000	0.000	0.000	−0.039	0.008
	Time	0.000	0.000	0.000	0.000	0.000	0.145
	Mode	0.000	0.000	0.000	0.000	0.000	0.000

^a^ PROBIT Model: PROBIT (p) = b + BX.

**Table 6:** Result of Weaken.

	Iteration	Find the Optimal Solution
Probit	38	Yes

**Table 7:** Test Result of Chi-Square.

		Chi-Square	df	Sig.
Probit	Pearson Fit Test	9621.558	2897	1.000
	Parallel Inspection	89.554	3	1.000

**Table 8:** Estimate of Respond Rate.

Variable	Control Group	Chi-Square	Estimate	Standard Deviation
	Number of Subjects	Number of Responses		
Probit	2973	589	19.81%	0.007

## Data Availability

The data used to support the findings of this study are available from the corresponding author upon request.
